# Outcome of incisional hernia repair in patients 80 years and older: results from the Herniamed-Registry

**DOI:** 10.1007/s10029-023-02866-0

**Published:** 2023-08-26

**Authors:** A. Mehdizadeh-Shrifi, C. Soll, R. N. Vuille-dit-Bille, F. Köckerling, D. Adolf, R. F. Staerkle

**Affiliations:** 1 Department of Surgery, University Hospital of Basel, Basel, Switzerland; 2 Ventravis – Practice for Abdominal Surgery, Dorfplatz 1, 6330 Cham, Switzerland; 3 University of Zurich, Zurich, Switzerland; 4 Department of Pediatric Surgery, University Children’s Hospital of Basel, Basel, Switzerland; 5 Hernia Center, Vivantes Humbold Hospital, Academic Teaching Hospital of Charité University Medicine, Berlin, Germany; 6 StatConsult GmbH, Magdeburg, Germany; 7 University of Lucerne, Lucerne, Switzerland

**Keywords:** Incisional hernia repair, Elderly patients, Complications, Risk factors

## Abstract

**Introduction:**

More and more often complex abdominal surgeries are performed in the elderly. Together with the ageing population these patients are at risk for incisional hernias. We aimed on assessing outcomes following incisional hernia surgery in patients 80 years and older.

**Material and methods:**

Using the Herniamed-Registry, a prospective multi-institutional database, data on patients undergoing surgery for incisional hernias were retrospectively assessed. 46,040 patients were included and divided by age. Intraoperative-, general-, and postoperative complications as well as 1-year follow-up outcomes were assessed and compared between patients 80 years and older vs younger than 80 years.

**Results:**

Intra- (2.3% vs 1.5%; *p* < 0.001) and postoperative (8.6% vs 7.2%; *p* = 0.001) complications, general complications (5.5% vs 3.0%; *p* < 0.001), as well as reoperations (3.8% vs 3.0%; *p* = 0.007) were more likely to occur in elderly patients. By contrast, recurrences (3.6% vs 4.5%; *p* = 0.007), pain at rest (7.3% vs 10.1%; *p* < 0.001) and on exertion (11.3% vs 18.3%; *p* < 0.001), as well as pain requiring treatment (5.4% vs 7.7%; *p* < 0.001) was less likely in the group of patients aged ≥ 80 years.

**Conclusion:**

Incisional hernia repair in patients 80 years and older is associated with a slightly higher complication risk but is quite acceptable and also have improved pain scores. The recurrence difference is also clinically unimportant.

## Introduction

Incisional hernias refer to a defect of the abdominal wall and are related to prior abdominal surgery, with midline incisional hernias being the most common subtype [1]. Incisional hernias are a frequent complication after abdominal wall incision [2]. In a systematic review and metaanalysis including 14,618 patients the prevalence of incisional hernias after midline incision was 12.8% (range: 0% to 35.6%) at a weighted mean of 23.7 months [3].

Despite the trend towards minimally invasive procedures in abdominal surgery, current literature estimates that at least two million laparotomies are still performed in the United States over the time span of one year [4]. As a consequence between 100,000 and 150,000 incisional hernia repairs are performed annually in the United States [5].

Higher age is a patient characteristic associated with increased incisional hernia rates [2, 3].

The United Nations estimates that the number of people aged 80 years and older will triple from 143 million in 2019 to 426 million in 2050 [6]. Additionally, the advancements in modern technology and surgery lead to a greater number of elderly undergoing complex surgeries, making them, consequently, vulnerable to the occurrence of incisional hernias postoperatively. Considering the massive changes in demographic culture, as well as the innovations of modern medicine, studying the elderly in relation to incisional hernias becomes even more important.

Despite the high incidence of incisional hernias, high data volumes on results following incisional hernia surgery in patients 80 years and older are lacking. We hence aimed on assessing outcomes (including intra-, postoperative- and general complications, reoperations, recurrences, and chronic pain) in patients aged ≥ 80 years.

## Material and methods

The Herniamed-Registry is a multicentre, online, hernia registry. It was founded in the year 2009 with the goal and emphasis to report on hernia surgery and respective outcome research. 836 participating hospitals and surgeons in private practice (Herniamed Study Group) in Germany, Austria and Switzerland, who have distributed patient data and outcomes following hernia surgery, are included. All patients signed informed consent and were informed about the fact that the respective hospital or practice should be informed about potential problems occurring postoperatively as well as requiring clinical control when necessary. After 1-, 5- and 10 years patients and their general practitioners are sent a questionnaire by the treating surgeon or hospital, enquiring once again about any postoperative complications. In addition, in the questionnaire patients and their general practitioners are asked about any pain at rest, pain on exertion or chronic pain requiring treatment. It is also asked about any suspicious protrusion.

The following retrospective analysis aims to compare and assess the prospective data collected for incisional hernias divided by age group ([< 80 years vs. ≥ 80 years] regarding intraoperative-, general- and postoperative complications, reoperation rate, recurrence, and chronic pain.

Analyses were performed using the software SAS 9.4 (SAS Institute Inc., Cary, NC, USA) and intentionally calculated to a full significance level of 5%, i.e. they were not corrected in respect of multiple tests, and each *p* value ≤ 0.05 represents a significant result.

**Fig. 1:**
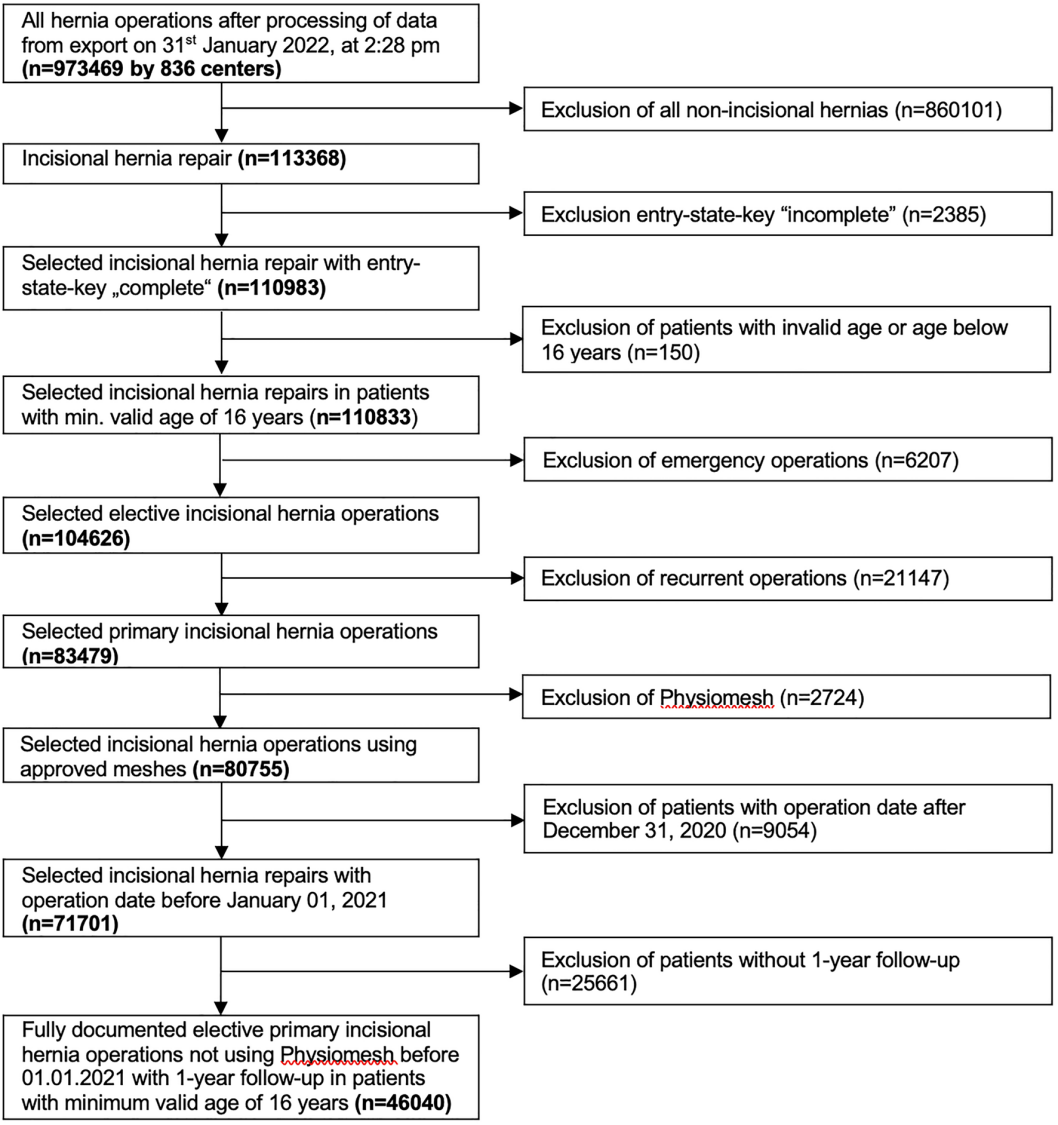
Flow chart for inclusion criteria

Amongst the 973,469 patients included in the database, the following items were used as inclusion criteria: surgery of an incisional hernia, complete documentation of the patient and respective surgery, patient age ≥ 16 years, no emergency surgery, no recurrent incisional hernias, usage of an approved mesh, exclusion of Physiomesh because of market retrieval, date of surgery until December 2020, available 1-year-follow-up data with complete documentation (Fig. 1).

Intraoperative complications included bleeding, organ injuries (i.e. injuries to vessels, bowel, bladder, stomach, spleen, liver etc.).

General complications included fever, urinary tract infections, diarrhoea, gastritis, thrombosis, pulmonary embolisms, pneumonia, chronic obstructive pulmonary disease (COPD), cardiac insufficiency, coronary heart disease, renal insufficiency, hypertensive crisis, and deceased patients.

Postoperative complications included bleeding, seroma formation, prolonged ileus, small bowel obstruction, bowel injury, anastomotic insufficiency, wound healing disorder and infection.

Unadjusted analysis was performed to analyse the association of specific parameters to the age groups. Chi-square test was used for categorical outcome variables and robust t-test (Satterthwaite) for continuous outcome variables with normal distribution. A binary logistic regression model was utilized to study the relation of patient and surgery-associated characteristics to the influencing variables. Pairwise odds ratios with 95% confidence interval are presented. Besides the age group, the following influencing parameters were assessed:

1. BMI [kg/m.^2^]
2. Gender [male/female]
3. ASA score [I/II/III-IV]
4. Defect size [W1 (< 4 cm)/ W2 ≥ 4-10 cm)/ W3 (> 10 cm)]
5. Surgical access [laparoscopic/open/other techniques not defined in the database]
6. EHS classification [medial/lateral/combined].^5^
7. Preoperative pain [yes/no/unknown]
8. Drain use [yes/no]
9. Mesh [yes/no]
10. Presence of risk factors [yes/no]

As well as postoperative complications for the analysis of pain at follow-up. Risk factors apply if at least one of the following risk factors are present:

1. Chronic obstructive pulmonary disease (COPD)
2. Diabetes mellitus
3. Aortic aneurysm
4. Immunosuppression
5. Corticosteroids
6. Smoking
7. Coagulopathy
8. Platelet aggregation inhibitors (discontinued less than 7 days before surgery)
9. Coumarin derivates (Quick/INR not in normal range).

## Results

### Unadjusted analysis

As seen in the patient inclusion flowchart (Fig. 1), 46,040 patients with primary elective incisional hernia repair were available for retrospective analysis of the prospectively collected data in the Herniamed-Registry. All included patients were at least 16 years old, and minimal follow-up was 1 year. 40,032 patients (91.29%) were in the age group < 80 years and 4008 patients (8.71%) were in the group ≥ 80 years.

The BMI in patients ≥ 80 years old was lower compared to the BMI in patients < 80 years (mean 27.2 ± 4.4 vs. 29.4 ± 5.9; *p* < 0.001). The group ≥ 80 years had more females (*p* < 0.001), higher ASA scores (*p* < 0.001), more middle-sized (vs. small-sized) defects (*p* < 0.001), were more likely to be treated with a drain (*p* < 0.005) and had more risk factors (*p* < 0.001) (Table 1).

**Table 1:** Results for the unadjusted tests between age group and the categorical variables

		Age group				
		< 80 years		> = 80 years		
		n	%	n	%	*p*
Gender	Male	21,533	51.2	1670	41.7	< 0.001
	Female	20,499	48.8	2338	58.3	
ASA PS	I	4905	11.7	85	2.1	< 0.001
	II	24,504	58.3	1602	40.0	
	III/IV	12,623	30.0	2321	57.9	
Type of access	Laparoscopic surgery	9869	23.5	932	23.3	0.054
	Open surgery	28,937	68.8	2809	70.1	
	Other	3226	7.7	267	6.7	
Defect size	I (< 4 cm)	16,425	39.1	1372	34.2	< 0.001
	II (4–10 cm)	18,567	44.2	1968	49.1	
	III (> 10 cm)	7040	16.7	668	16.7	
EHS classification	Medial	31,869	75.8	2879	71.8	< 0.001
	Lateral	6899	16.4	795	19.8	
	Combined	3264	7.8	334	8.3	
Preoperative pain	No	13,999	33.3	1340	33.4	0.985
	Yes	24,628	58.6	2343	58.5	
	Unknown	3405	8.1	325	8.1	
Drainage	Yes	23,674	56.3	2349	58.6	0.005
	No	18,358	43.7	1659	41.4	
Mesh	Yes	36,822	87.6	3550	88.6	0.075
	No	5210	12.4	458	11.4	
Risk factors—total	Yes	16,566	39.4	1884	47.0	< 0.001
	No	25,466	60.6	2124	53.0	
COPD	Yes	4045	9.6	458	11.4	< 0.001
	No	37,987	90.4	3550	88.6	
Diabetes	Yes	5162	12.3	562	14.0	0.001
	No	36,870	87.7	3446	86.0	
Aortic aneurysm	Yes	631	1.5	81	2.0	0.011
	No	41,401	98.5	3927	98.0	
Immunosuppression	Yes	838	2.0	48	1.2	< 0.001
	No	41,194	98.0	3960	98.8	
Corticoids	Yes	656	1.6	89	2.2	0.002
	No	41,376	98.4	3919	97.8	
Smoking	Yes	5432	12.9	121	3.0	< 0.001
	No	36,600	87.1	3887	97.0	
Coagulopathy	Yes	823	2.0	129	3.2	< 0.001
	No	41,209	98.0	3879	96.8	
Antithrombotic medication	Yes	4728	11.2	832	20.8	< 0.001
	No	37,304	88.8	3176	79.2	
Anticoagulant medication	Yes	1083	2.6	316	7.9	< 0.001
	No	40,949	97.4	3692	92.1	

The unadjusted analysis showed more intra- (*p* < 0.001), postoperative (*p* < 0.001) and general (*p* < 0.001) complications, as well as more reoperations (*p* = 0.007) in elderly patients. By contrast, recurrences (*p* = 0.007), pain at rest (*p* < 0.001) and on exertion (*p* < 0.001), as well as pain requiring treatment on 1-year follow-up (*p* < 0.001) were less likely in the group of patients aged ≥ 80 years (Table 2).

**Table 2:** Results for the unadjusted tests between age group and influencing variables

		Age group				
		< 80 years		> = 80 years		
		n	%	n	%	*p*
Intraoperative complications—total	Yes	623	1.5	91	2.3	< 0.001
	No	41,409	98.5	3917	97.7	
General complications—total	Yes	1277	3.0	219	5.5	< 0.001
	No	40,755	97.0	3789	94.5	
Postoperative complications—total	Yes	3030	7.2	344	8.6	0.001
	No	39,002	92.8	3664	91.4	
Complication-related reoperations	Yes	1277	3.0	153	3.8	0.007
	No	40,755	97.0	3855	96.2	
Recurrence on 1-year follow-up	Yes	1909	4.5	145	3.6	0.007
	No	40,123	95.5	3863	96.4	
Pain on exertion on 1-year follow-up	Yes	7680	18.3	452	11.3	< 0.001
	No	34,352	81.7	3556	88.7	
Pain at rest on 1-year follow-up	Yes	4252	10.1	291	7.3	< 0.001
	No	37,780	89.9	3717	92.7	
Pain requiring treatment on 1-year follow-up	Yes	3247	7.7	217	5.4	< 0.001
	No	38,785	92.3	3791	94.6	

### Multivariable analyses

#### Intraoperative complications

The risk of intraoperative complications was significantly associated with defect size (*p* < 0.001), use of a drain (*p* < 0.001; OR 2.5), laparoscopic surgery (*p* < 0.001), use of mesh (*p* < 0.001), age ≥ 80 years (*p* = 0.002), and female gender (*p* = 0.010; OR 1.2).

Given the prevalence of intraoperative complications of 1.56%, the OR of 1,445 for the age group over 80 years corresponds to one intraoperative complication in 19 cases out of 1000 operations in patients over 80 years compared to 13 cases out of 1000 operations in patients under 80 years (Table 3).

**Table 3:**
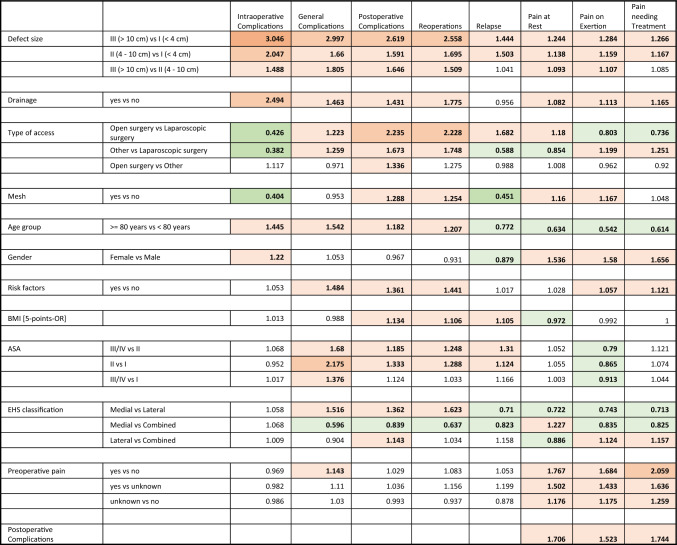
Multivariate analysis (Color figure online)

Given are the odds ratios (ORs). Color code: OR 3.0–3.999 dark orange; OR 2.0–2.999 orange; OR 1.0–1.999 light orange; OR 0.5–0.999 light green; OR 0.333–0.499 green. Numbers in bold: *p* < 0.05

#### Postoperative complications

A larger defect (*p* < 0.001), an open surgical procedure (*p* < 0.001), a higher BMI (*p* < 0.001), the presence of at least one risk factor (*p* < 0.001), the (need for) use of drains (*p* < 0.001), a higher ASA score (*p* < 0.001), the insertion of a mesh (*p* < 0.001) and older age (*p* = 0.007) led to an increased risk of postoperative complications.

In the EHS classifications, there was a lower postoperative complication rate, especially for lateral hernias (*p* < 0.001).

The relation to the age group (OR = 1.182), corresponds to approximately 79 postoperative complications out of 1000 hernias in patients over 80 years compared to 68 cases out of 1000 in patients under 80 years, with an overall prevalence of 7.31% (Table 3).

##### Reoperations

The risk of reoperations was significantly associated with a larger defect size (*p* < 0.001), open surgery (*p* < 0.001), the use of a drain (*p* < 0.001), the presence of risk factors (*p* < 0.001), the EHS classification (with lateral hernias being associated with a lower complication-related reoperation rate) (*p* < 0.001), higher BMI (*p* < 0.001), higher ASA score (*p* < 0.001), older age (*p* = 0.039) and use of a mesh (*p* = 0.050).

Comparing the age groups (OR = 1.207), this would correspond to 34 reoperations in 1000 operations in the group of patients over 80 years of age compared to 28 reoperations in 1000 operations in the group under 80 years of age (prevalence of reoperations: 3.09%) (Table 3).

#### General complications

The overall complication risk was significantly related to a larger defect size (*p* < 0.001), a higher ASA classification (*p* < 0.001), the presence of risk factors (*p* < 0.001), age ≥ 80 years (*p* < 0.001), the need for drains (*p* < 0.001), the EHS classification (with lateral hernias in particular leading to a lower complication rate) (*p* < 0.001) and open surgery (*p* = 0.014) (Table 3).

The relation to the age group (OR = 1.542) corresponds to about 39 general complications in 1000 operations in the group of patients over 80 compared to 26 cases in 1000 operations in the group of patients under 80 years (prevalence 3.25%).

#### Recurrence

Mesh insertion (*p* < 0.001), medial EHS classification (*p* < 0.001), ‘other’ surgical procedures (*p* < 0.001), and older age (*p* = 0.004) and female gender (*p* = 0.005) were associated with a lower risk of recurrence. Larger defects (*p* < 0.001), a higher BMI (*p* < 0.001), and a higher ASA score (*p* = 0.006) were associated with a higher risk of recurrence.

The demonstrated benefit in elderly patients (overall prevalence = 4.46%, OR = 0.772) corresponds to approximately 39 recurrences per 1000 hernias in the over-80 age group compared to 50 recurrences per 1000 in the under-80 age group (Table 3).

#### Pain at rest

Preoperative pain (*p* < 0.001), postoperative complications (*p* < 0.001), lateral hernias (*p* < 0.001), larger defects (*p* < 0.001), female gender (*p* < 0.001), mesh insertion (*p* = 0.008), and the use of a drain (*p* = 0.050) were associated with pain at rest one year after surgery.

Older age (*p* < 0.001), ‘other’ surgical procedures (*p* = 0.031) and a higher BMI (*p* = 0.042) were associated with lower risk of pain at rest during follow-up.

This corresponds to a risk of pain at rest on follow-up in 79 out of 1000 patients in the group of patients over 80 years compared to 119 out of 1000 patients in the group under 80 years (prevalence 9.86%) (Table 3).

#### Pain on exertion

Preoperative pain (*p* < 0.001), postoperative complications (*p* < 0.001), larger defects (*p* < 0.001), use of mesh (*p* < 0.001) and drainage (*p* < 0.001) and the presence of at least one risk factor (*p* = 0.039) led to an increase in the risk of pain on exertion one year after surgery. The risk of stress-related pain was also increased in females (*p* < 0.001). Older age (*p* < 0.001), medial hernias (*p* < 0.001), a higher ASA score (*p* < 0.001), and ‘other’ surgical procedures (*p* < 0.001) were associated with lower risk of pain on exertion.

Regarding age groups, this corresponds to an incidence of exertional pain at follow-up in 133 out of 1000 patients in the over-80 s group compared to 220 out of 1000 patients in the under-80 s group (prevalence 17.65%) (Table 3).

#### Chronic pain requiring treatment

Preoperative pain (*p* < 0.001), postoperative complications (*p* < 0.001), larger defects (*p* < 0.001), use of drains (*p* < 0.001), female gender (*p* < 0.001), and the presence of at least one risk factor (*p* = 0.003) were associated with chronic pain requiring treatment.

Medial hernias (*p* < 0.001), older age (*p* < 0.001) and ‘other’ surgical procedures (*p* < 0.001) were associated with a reduced risk of pain requiring treatment.

Regarding age groups, this result would correspond to an occurrence of pain requiring treatment in the follow-up in 58 cases out of 1000 operations in the group over 80 years compared to 92 cases out of 1000 operations in the group of patients under 80 years (prevalence 7.52%) (Table 3).

## Discussion

Considering the aging population, together with the increase in surgical therapies, incisional hernias represent an incremental health care burden [2]. The analysis of the association of age group (< 80 years versus ≥ 80 years) to general, intraoperative and postoperative complication, reoperation, recurrence, and chronic pain was conscientiously studied in 46,040 well documented cases of incisional hernia repairs performed between 05.01.2009 and 31.12.2020 with a minimal follow-up period of one year. Analyzing 46,046 patients with incisional hernias, we observed more intra-, general and postoperative complications, as well as reoperations in the elderly. But the complication risk is only slightly higher and quite acceptable. Furthermore, analyzing patients one year after surgery for incisional hernia, elderly patients showed a lower recurrence rate and less chronic pain.

As outlined by the group of Caglia et al., especially, patients 80 years and older are associated with several comorbidities and higher ASA scores, which, consequently, lead to more severe intra -and postoperative complications [7]. In this regard, our data outlines the representation of risk factors in this age group making patients 80 years and older a specifically vulnerable patient collective.

Considering the vulnerability of this group, the optimal surgical approach is of great importance for patients 80 years and older. While the risk for intraoperative complications was increased via a laparoscopic approach, both general – and postoperative complications were higher when operated openly, an observation that is in line with data from others [7]. This is also underlined by Aly et al. who emphasized on the surgical approach for ventral hernias in the elderly population [8]. While the open approach still remains the most prevalent, a laparoscopic approach has shown to be safe with lower risk of infection, reoperation, and morbidity [8]. The feasibility and benefits for the geriatric population are further exemplified by Elhage et al., who continue to show the benefits for the elderly. Despite the rates of comorbidities and large hernia defects, laparoscopic ventral hernia repair can be performed in the geriatric patient [9]. While the optimal surgical approach for incisional hernias in patients 80 years and older remains debated, our results contribute to the important discussion for the treatment of incisional hernias and emphasize the necessity of an individualized approach for the elderly patient. The surgical approach should, therefore, be critically reviewed, considering the vulnerability of this group.

Another important factor is the relation of intraoperative, general and postoperative complications with the use of drains. This could be correlated with the use of drains in patients with larger defect sizes. Luo et al. were able to show that post-operative wound infection rate and overall complications may increase when drains were used, especially in complex patients with risk factors [10]. Since risk factors and a large defect size where overwhelmingly present in patients 80 years and older, the use of drains should be considered carefully for this vulnerable patient group.

Furthermore, it is also necessary to review the implementation of mesh repair in incisional hernia. Luijendijk et al. were able to show the superiority of mesh repair as compared to suture repair, irrespective of defect size, making it the standard treatment option [11]. However, our data shows that 11.4% of patients 80 years and older did not receive mesh repair. Furthermore, analyzing patients one year after surgery for incisional hernia, elderly patients showed lower recurrence rate and postoperative pain.

This has also been shown by Staerkle et al. who outlined younger age as an independent risk factor for postoperative pain following hernia surgery. Age presented with an inverse relation with pain[12]. This inverse age-pain relationship has been exemplified by various studies, as well as recognized by the European Hernia Society [13–16].

Aasvang et al. explained the inverse age-pain relationship may be related to the decreased sensory function [17]. Pierides et al., consequently, demonstrated that the reduced postoperative pain in the elderly is a factor rather supporting an operative approach [18].

As the present study is based on a registry, there are certain possible confounding factors: Participation in the registry is voluntary. Therefore, it may be that surgeons with a special interest in hernia surgery are more likely to participate. Missing or incorrect data may further limit a registry, despite detailed documentation. However, the Herniamed-Registry focuses on certain aspects to advance the data collection. These include surgeon responsibility for correctness of used data, review of perioperative outcome on 1-year follow-up and meticulous control of data entry, as well as indication of missing data by the applied software. Another limitation is that the indication for surgery and the choice of the technique is different between the participating hospitals and surgeons in the Herniamed Registry.

## Conclusion

In conclusion, our study shows an acceptable complication risk for patients 80 years and older with incisional hernia repairs. Considering the lower rate of postoperative chronic pain und recurrence in the elderly, the surgical approach should further reduce the rate of perioperative complications.

We, therefore, recommend meticulous pre-surgical work up for this respective patient group and assessment of all potential risk factors. And the procedure should be done by a surgeon with adequate experience in hernia surgery. Further studies will be required to fully understand the interplay between age and incisional hernia repair.

## Data Availability

Not applicable.
